# Lower number of plasmacytoid dendritic cells in peripheral blood of children with bronchiolitis following respiratory syncytial virus infection

**DOI:** 10.1111/irv.12242

**Published:** 2014-02-17

**Authors:** Kaizhi Weng, Jiaxiang Zhang, Xuqiao Mei, Ayang Wu, Baozhong Zhang, Mengyun Cai, Yuanhai Zheng, Zhuanye Ke

**Affiliations:** a Department of Pediatrics, Zhangzhou Municipal Hospital Affiliated to Fujian Medical UniversityZhangzhou, China; b Department of Laboratory Medicine, Zhangzhou Municipal Hospital Affiliated to Fujian Medical UniversityZhangzhou, China

**Keywords:** Bronchiolitis, dendritic cells, respiratory syncytial virus

## Abstract

**Objectives:**

Dendritic cells (DCs) are key mediators of allergic airway inflammation. Thus, it is important to understand the relationship between respiratory syncytial virus (RSV) infection and DCs, especially in children with RSV bronchiolitis.

**Methods:**

We collected peripheral blood from 71 children with RSV bronchiolitis at the time of admission and 28 children who were followed up 3 months following admission. Flow cytometry was performed to detect dendritic cell immunophenotypes.

**Results:**

Patients with RSV bronchiolitis exhibited significantly higher number of myeloid DCs and lower number of plasmacytoid DCs at the time of admission and 3 months following discharge, compared with healthy controls. These children had a significantly higher myeloid/plasmacytoid ratio 3 months after discharge compared with healthy controls.

**Conclusions:**

Among children with RSV bronchiolitis, there is an imbalance in peripheral blood myeloid/plasmacytoid ratio. The low number of plasmacytoid DCs in peripheral blood indicates the development of bronchiolitis due to RSV infection.

## Introduction

Bronchiolitis is a disorder most commonly resulting from a lower respiratory tract viral infection, especially by respiratory syncytial virus (RSV).^1^ Bronchiolitis is a common cause of hospitalization in infants and young children, resulting in a large disease burden worldwide.^2^,^3^ By the second year of life, 90% of children can be infected by RSV at least once. Respiratory syncytial virus infection in infants is associated with lower respiratory tract viral infection (bronchiolitis and pneumonia) in 25–40% of cases with hospitalization rates of 0·5–3% (highest in infants <6 months of age).^2^

Dendritic cells (DCs) are antigen-presenting cells, which can stimulate naïve T cells and initiate primary immune responses.^4^ In humans, two major subpopulations of DCs exist: myeloid DCs (mDCs) and plasmacytoid DCs (pDCs). Myeloid DCs can secrete interleukin (IL)-12, a potent Th1-driving factor, and are often viewed as Th1-promoting antigen-presenting cells (APCs).^5^ In animal models of allergic asthma, the induction and maintenance of airway inflammation is primarily a function of mDCs.^6^,^7^ In contrast, tolerance to inhaled allergens is likely a function of pDCs.^8^ Children diagnosed with asthma following severe RSV bronchiolitis appeared to have a relative deficiency of plasmacytoid DCs in peripheral blood.^9^ Approximately 50% of children who experience severe RSV bronchiolitis have a subsequent asthma diagnosis.^10^

Given the critical role of DCs in the initiation of immune responses, it is important to understand the relationship between RSV infection and DCs. The majority of studies on bronchiolitis have focused on DCs in animal models and shown that RSV infection results in sustained increase in the number of mature dendritic cells in the lung.^11^ However, very little is known about the interaction between RSV and the DC system, particularly in human peripheral blood DC subsets. Therefore, in this study, we analyzed mDC and pDC subsets from hospitalized children with bronchiolitis caused by RSV infection.

## Materials and methods

### Patient population

Inclusion criteria for enrollment into RSV group were as follows: age <18 months, illness requiring hospitalization, RSV infection, and diagnoses of bronchiolitis. Bronchiolitis was diagnosed according to the presence of an acute respiratory tract infection characterized by respiratory distress (tachypnea, use of accessory muscles), cough, widespread crackles, wheezing associated with signs of viral infection (coryza).^12^ All patients were hospitalized for bronchiolitis at the Department of Pediatrics, Zhangzhou Municipal Hospital Affiliated to Fujian Medical University, from November 1, 2011, to June 31, 2012. The first consent interview took place soon after admission. During this interview, parents were provided with written information. A second interview was scheduled when patients were discharged 3 months after admission, during which additional information was provided and informed consent was obtained. The control population consisted of healthy infants with no history of upper respiratory illness within the preceding 3 weeks. These children were identified in the child healthcare department while undergoing physical health examinations. Polymerase chain reaction (PCR) tests of nasal wash samples as described previously^13^ were performed at enrollment for all healthy control children and for all patients with RSV infection who had undergone follow-up evaluations. Exclusion criteria for all groups were as follows: age >15 months, immunosuppressive medication, recurrent (more than one) wheezing episode, a history of asthma, and history of chronic lung disease or congenital diseases. Informed consent was obtained from the parents of all patients at enrollment in the study, and the institutional review board at the Fujian Medical University approved the study.

### Flow cytometrical identification of dendritic cells

Dendritic cells were identified according to the previous study^14^ with some modification. Briefly, DCs were identified in heparinized whole-blood samples using a combination of fluorochrome-conjugated monoclonal antibodies to different membrane markers. Myeloid DCs were lin^neg^CD11c^high^CD123^low^. Plasmacytoid DCs were lin^neg^CD11c^neg^CD123^high^. Whole-blood samples of each case were divided into four tubes. Reagents were obtained from BD Biosciences: to sample A, 20 μl of lineage cocktail 1-fluorescein isothiocyanate (Lin1-FITC), containing a mixture of anti-CD3, anti-CD14, anti-CD16, anti-CD56, anti-CD19, and anti-CD20 antibodies, 20 μl of anti-HLA-DR-PerCP, and 20 μl of mouse anti-human IgG2a-PE were added; to sample B, 20 μl of Lin1-FITC, 20 μl of anti-HLA-DR-PerCP, and 20 μl of anti-CD11c-PE were added; to sample C, 20 μl of Lin1-FITC, 20 μl of anti-HLA-DR-PerCP, and 20 μl of mouse anti-human IgG1-PE were added; to sample D, 20 μl of Lin1-FITC, 20 μl of anti-HLA-DR-PerCP, and 20 μl f-CD123-PE were added. All samples were incubated for 45 minutes at 4°C in the dark. Cells were fixed and lysed for 10 minutes at room temperature using FACS lysing solution (BD Bioscience, San Jose, CA, USA). Cells were spun at 200 ***g*** for 10 minute, washed twice with phosphate-buffered saline (PBS), and then resuspended in 0·2 ml of PBS. Data acquisition was performed on a Beckman coulter (Beckman Coulter, Inc., Fullerton, CA, USA) using a well-defined gating strategy in the Cell Quest Pro software. At least 1 × 10^5^ cells for each sample were analyzed.

### Statistical analysis

Statistical analyses were performed using the spss version 18.0 software for Windows (SPSS Inc., Chicago, IL, USA). Results were expressed as mean and standard deviation (SD) and analyzed using the independent samples *t-*test or the paired samples *t-*test or chi-square test. All data were analyzed using a homogeneity test for variance. A *P*-value < 0·05 was considered significant.

## Results

### Patient enrollment

From November 1, 2011 to June 31, 2012, seventy-one patients with acute bronchiolitis due to RSV infection were eligible for study participation. Of these, twenty-eight patients agreed to a follow-up 3 months following admission. Baseline characteristics did not differ between follow-up patients and non-follow-up patients (Table1). Forty-eight healthy infants were also enrolled during the same period. No significant differences in demographic data were observed between the two groups (Table2). All patients with acute bronchiolitis due to RSV infection were diagnosed according to the symptoms of bronchiolitis requiring hospitalization by pediatric respiratory specialists.

**Table 1:** The demographics of follow-up and non-follow-up patients

Patients	Follow-up patients (*n* = 28)	Patients not followed up (*n* = 43)	*P*
Age (months) median (range)	7·25 (2–16)	6·34 (3–17)	0·259
Gender M/F, *n* (%)	20 (71·4)/8 (28·6)	37 (86·1)/6 (13·9)	0·130
mDCs (cells per 10^5^ PBNC)	567 ± 391	551 ± 296	0·850
pDCs (cells per 10^5^ PBNC)	160 ± 799	190 ± 177	0·417

PBNC, peripheral blood nucleated cells.

**Table 2:** The demographics of patients and healthy controls

Patients	Follow-up patients (*n* = 28)	Healthy infants (*n* = 48)	*P*
Age (months) median (range)	7·25 (2–16)	6·17 (2–15)	0·149
Gender M/F, *n* (%)	20 (71·4)/8 (28·6)	28 (58·3)/20 (41·7)	0·254

### Comparison of DCs between patients with RSV bronchiolitis at admission and healthy controls

**Figure 1:**
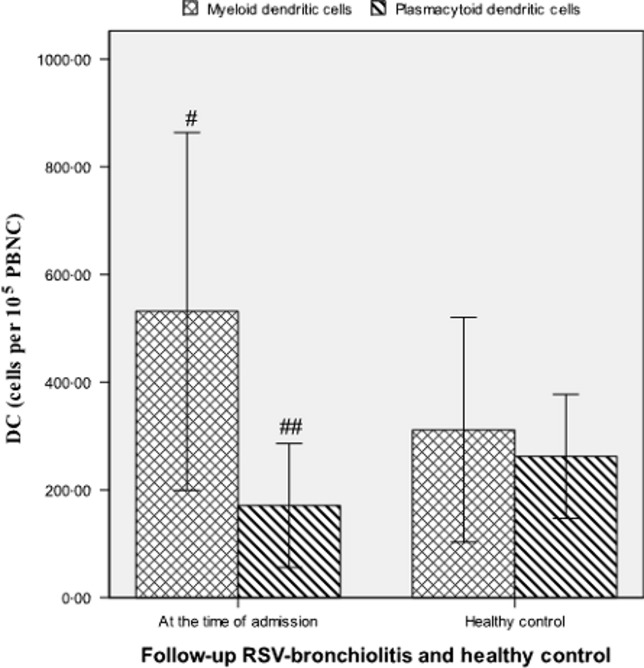
Quantities of circulating dendritic cells per 10^5^ peripheral blood nucleated cells in follow-up patients with respiratory syncytial virus bronchiolitis at the time of admission and healthy controls. ^#^*P* < 0·01 versus healthy controls, ^##^*P* < 0·01 versus healthy controls.

Follow-up patients with RSV bronchiolitis at the time of admission had a significantly higher quantity of peripheral mDCs compared with healthy controls (532 ± 332 versus 311 ± 209 cells per 10^5^ peripheral blood nucleated cells (PBNC), *P* = 0·001) (Figure1). However, children (follow-up) with RSV bronchiolitis at the time of admission exhibited significantly lower quantities of pDCs compared with healthy controls (171 ± 115 versus 262 ± 115 cells per 10^5^ PBNC, *P* = 0·001) (Figure1). The myeloid/plasmacytoid ratio was significantly higher than the healthy controls (3·875 ± 2·429 versus 1·371 ± 1·002, *P* = 0·002).

### Comparison of DCs in patients with RSV bronchiolitis between admission and follow-up

**Figure 2:**
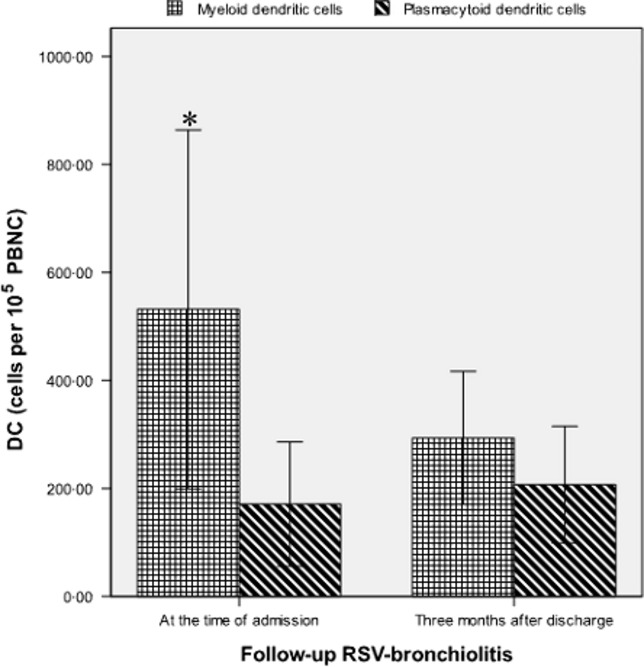
Quantities of peripheral dendritic cells per 10^5^ peripheral blood nucleated cells in follow-up patients with respiratory syncytial virus bronchiolitis at the time of admission and 3 months following discharge. *P < 0·01 versus 3 months after discharge.

For patients with RSV bronchiolitis who were followed up, they had a significantly higher quantity of peripheral mDCs at the time of admission than at 3 months after discharge (532 ± 332 versus 294 ± 123 cells per 10^5^ PBNC, *P* = 0·001) (Figure2), but had lower quantities of pDCs than at 3 months after discharge (171 ± 115 versus 207 ± 108 cells per 10^5^ PBNC, *P* = 0·236) (Figure2). In addition, the myeloid/plasmacytoid ratio was significantly higher at the time of admission than at 3 months after discharge (3·875 ± 2·429 versus 2·020 ± 1·807, *P* = 0·002).

### Comparison of DCs between patients with RSV bronchiolitis at follow-up and healthy controls

**Figure 3:**
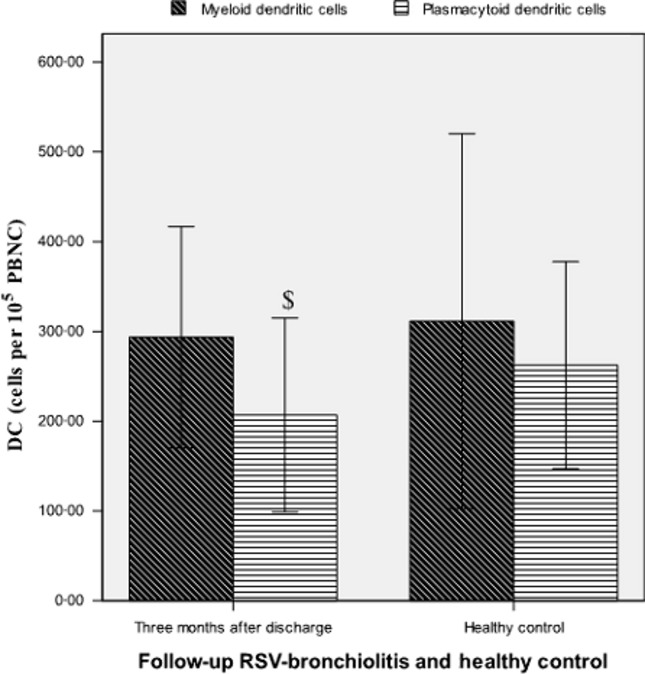
Quantities of peripheral dendritic cells per 10^5^ peripheral blood nucleated cells in follow-up patients with respiratory syncytial virus bronchiolitis 3 months following discharge and healthy controls. ^$^*P* < 0·05 versus healthy control.

There was no significant difference in the quantity of mDCs in patients with RSV bronchiolitis at 3 months after discharge, compared with healthy controls (mDCs 294 ± 123 versus 311 ± 209 cells per 10^5^ PBNC, *P* = 0·639) (Figure3). However, children with RSV bronchiolitis at 3 months after discharge exhibited significantly lower quantities of pDCs compared with healthy control (pDCs 207 ± 108 versus 262 ± 115 cells per 10^5^ PBNC, *P* = 0·039) (Figure3). The myeloid/plasmacytoid ratio was significantly higher than in patients at 3 months after discharge than in healthy controls (2·020 ± 1·807 versus 1·371 ± 1·002, *P* = 0·047).

## Discussion

Respiratory syncytial virus is the most common cause of childhood acute lower respiratory infection.^2^ Respiratory syncytial virus is also an important cause of death in childhood from acute lower respiratory infection, indicating that it is not efficiently controlled by the immune system. Dendritic cells are known to play a central role in sensing the presence of foreign antigens and infectious agents and initiating appropriate immune responses against them.^15^

In the present study, we found that children diagnosed with RSV bronchiolitis exhibited higher quantities of mDCs compared with that of healthy controls at the time of admission. Interestingly, we also found that patients with RSV bronchiolitis who were followed up 3 months later had higher number of mDCs at the time of admission compared with 3 months after discharge. Myeloid DCs can induce Th1 differentiation *in vitro*. Children suffering from RSV infection had high levels of Th1 cytokines.^16^–^18^ Th1 immune response is necessary for protective response against RSV.^18^ T cells cultured with mDCs have been shown to secrete large amounts of interferon-γ (IFN-γ).^19^ Interferon-γ binds to specific surface receptors on primary target cells and induces the transcription of a variety of genes that mount antiviral response.^20^ This antiviral immune response at an early stage of viral infection may explain our observation of increased mDCs in patients with RSV bronchiolitis at the time of admission.

Our data showed that patients with RSV bronchiolitis at the time of admission had significantly lower numbers of pDCs than healthy controls. Gill *et al*.^21^ observed that infection with RSV and other respiratory viruses mobilized pDCs to the site of viral entry. This may explain the decrease in pDCs in the peripheral blood of children with RSV bronchiolitis. Our findings are consistent with previous studies comparing DC levels between asthmatics and healthy subjects.^14^,^22^ de Heer *et al*.^8^ showed that pDCs provided intrinsic protection against inflammatory responses to “harmless” antigens. Plasmacytoid DC counts were lower in allergic children, especially in asthmatics compared with healthy controls.^14^ In the present study, we found that children with RSV bronchiolitis who were followed up 3 months after discharge exhibited significantly lower quantities of pDCs compared with healthy controls. Silver *et al*.^9^ recently reported that children diagnosed with asthma after severe RSV bronchiolitis appeared to have a deficiency of plasmacytoid DCs in peripheral blood. Based on these data, we hypothesized that low quantities of pDCs in children diagnosed with RSV bronchiolitis indicate their susceptibility to asthma. Further clinical studies are needed to verify this hypothesis.

Interestingly, we found that patients with RSV bronchiolitis who were followed up exhibited significantly higher myeloid/plasmacytoid ratios at the time of admission and 3 months after discharge, compared with healthy controls. These results indicate that there is an imbalance in circulating dendritic cells in patients with bronchiolitis caused by RSV infection.

In summary, among children with RSV bronchiolitis, there appears to be an imbalance in the peripheral blood myeloid/plasmacytoid ratio. The immune imbalance of DCs in children diagnosed with RSV bronchiolitis is similar to that in children suffering from asthma. Further studies of DCs in RSV-associated bronchiolitis may provide better approaches for the diagnosis and therapy of bronchial asthma following RSV infection.

## References

[1] Wright M, Piedimonte G (2011). Respiratory syncytial virus prevention and therapy: past, present, and future. Pediatr Pulmonol.

[2] Nair H, Nokes DJ, Gessner BD (2010). Global burden of acute lower respiratory infections due to respiratory syncytial virus in young children: a systematic review and meta-analysis. Lancet.

[3] Učakar V, Sočan M, Trilar KP (2013). The impact of influenza and respiratory syncytial virus on hospitalizations for lower respiratory tract infections in young children: Slovenia, 2006-2011. Influenza Other Respi Viruses.

[4] Banchereau J, Steinman RM (1998). Dendritic cells and the control of immunity. Nature.

[5] Lambrecht BN, Hammad H (2003). Taking our breath away: dendritic cells in the pathogenesis of asthma. Nat Rev Immunol.

[6] Lambrecht BN, De Veerman M, Coyle AJ (2000). Myeloid dendritic cells induce Th2 responses to inhaled antigen, leading to eosinophilic airway inflammation. J Clin Invest.

[7] van Rijt LS, Jung S, KleinJan A (2005). *In vivo* depletion of lung CD11c1 dendritic cells during allergen challenge abrogates the characteristic features of asthma. J Exp Med.

[8] de Heer HJ, Hammad H, Soullie T (2004). Essential role of lung plasmacytoid dendritic cells in preventing asthmatic reactions to harmless inhaled antigen. J Exp Med.

[9] Silver E, Yin-DeClue H, Schechtman KB (2009). Lower levels of plasmacytoid dendritic cells in peripheral blood are associated with a diagnosis of asthma 6 yr after severe respiratory syncytial virus bronchiolitis. Pediatr Allergy Immunol.

[10] Bacharier LB, Cohen R, Schweiger T (2012). Determinants of asthma after severe respiratory syncytial virus bronchiolitis. J Allergy Clin Immunol.

[11] Beyer M, Bartz H, Horner K (2004). Sustained increases in numbers of pulmonary dendritic cells after respiratory syncytial virus infection. J Allergy Clin Immunol.

[12] Smyth RL, Openshaw PJ (2006). Bronchiolitis. Lancet.

[13] Mahony JB (2008). Detection of respiratory viruses by molecular methods. Clin Microbiol Rev.

[14] Hagendorens MM, Ebo DG, Schuerwegh AJ (2003). Differences in circulating dendritic cell subtypes in cord blood and peripheral blood of healthy and allergic children. Clin Exp Allergy.

[15] Gill MA (2012). The role of dendritic cells in asthma. J Allergy Clin Immunol.

[16] Brandenburg AH, Kleinjan A, van Het Land B (2000). Type 1-like immune response is found in children with respiratory syncytial virus infection regardless of clinical severity. J Med Virol.

[17] van Schaik SM, Tristram DA, Nagpal IS (1999). Increased production of IFN-gamma and cysteinyl leukotrienes in virus-induced wheezing. J Allergy Clin Immunol.

[18] Cautivo KM, Bueno SM, Cortes CM (2010). Efficient lung recruitment of respiratory syncytial virus-specific Th1 cells induced by recombinant bacillus Calmette-Guerin promotes virus clearance and protects from infection. J Immunol.

[19] Rissoan MC, Soumelis V, Kadowaki N (1999). Reciprocal control of T helper cell and dendritic cell differentiation. Science.

[20] Goodbourn S, Didcock L, Randall RE (2000). Interferons: cell signalling, immune modulation, antiviral response and virus countermeasures. J Gen Virol.

[21] Gill MA, Palucka AK, Barton T (2005). Mobilization of plasmacytoid and myeloid dendritic cells to mucosal sites in children with respiratory syncytial virus and other viral respiratory infections. J Infect Dis.

[22] Upham JW, Rate A, Kusel M, Sly PD, Johnston SL, Holt PG (2006). The frequencies of plasmacytoid and myeloid dendritic cell subsets in infancy are differentially associated with risk for viral respiratory infections, allergic sensitization and asthma. Proc Am Thorac Soc.

